# Excision Repair Cross-Complementation Group 1 (ERCC1) Status and Lung Cancer Outcomes: A Meta-Analysis of Published Studies and Recommendations

**DOI:** 10.1371/journal.pone.0025164

**Published:** 2011-10-14

**Authors:** Richard A. Hubner, Richard D. Riley, Lucinda J. Billingham, Sanjay Popat

**Affiliations:** 1 Department of Medical Oncology, Christie NHS Foundation Trust, Manchester, United Kingdom; 2 Department of Public Health, Epidemiology and Biostatistics, University of Birmingham, Birmingham, United Kingdom; 3 Cancer Research UK Clinical Trials Unit, University of Birmingham, Birmingham, United Kingdom; 4 MRC Midland Hub for Trials Methodology Research, University of Birmingham, Birmingham, United Kingdom; 5 Department of Medicine, Royal Marsden NHS Foundation Trust, London, United Kingdom; 6 Molecular Genetics Group, Imperial College, London, United Kingdom; Institut Gustave Roussy, France

## Abstract

**Purpose:**

Despite discrepant results on clinical utility, several trials are already prospectively randomizing non-small cell lung cancer (NSCLC) patients by ERCC1 status. We aimed to characterize the prognostic and predictive effect of ERCC1 by systematic review and meta-analysis.

**Methods:**

Eligible studies assessed survival and/or chemotherapy response in NSCLC or SCLC by ERCC1 status. Effect measures of interest were hazard ratio (HR) for survival or relative risk (RR) for chemotherapy response. Random-effects meta-analyses were used to account for between-study heterogeneity, with unadjusted/adjusted effect estimates considered separately.

**Results:**

23 eligible studies provided survival results in 2,726 patients. Substantial heterogeneity was observed in all meta-analyses (I^2^ always >30%), partly due to variability in thresholds defining ‘low’ and ‘high’ ERCC1. Meta-analysis of unadjusted estimates showed high ERCC1 was associated with significantly worse overall survival in platinum-treated NSCLC (average unadjusted HR = 1.61, 95%CI:1.23–2.1, p = 0.014), but not in NSCLC untreated with chemotherapy (average unadjusted HR = 0.82, 95%CI:0.51–1.31). Meta-analysis of adjusted estimates was limited by variable choice of adjustment factors and potential publication bias (Egger's p<0.0001). There was evidence that high ERCC1 was associated with reduced response to platinum (average RR = 0.80; 95%CI:0.64–0.99). SCLC data were inadequate to draw firm conclusions.

**Conclusions:**

Current evidence suggests high ERCC1 may adversely influence survival and response in platinum-treated NSCLC patients, but not in non-platinum treated, although definitive evidence of a predictive influence is lacking. International consensus is urgently required to provide consistent, validated ERCC1 assessment methodology. ERCC1 assessment for treatment selection should currently be restricted to, and evaluated within, clinical trials.

## Introduction

Lung cancer is the commonest cause of cancer death globally, accounting for around 1.3 million deaths per year [1]. Despite advances in therapeutics, survival from both major subtypes (non-small cell lung cancer (NSCLC) and SCLC) remain poor, with only around 5% of all patients reaching 5 years. Standard of care for both advanced NSCLC and SCLC is platinum-based doublet chemotherapy, with non-platinum doublets inferior [2], [3]. In NSCLC platinum doublets are associated with response rates of 25–30% [4]. A number of tumour biomarkers have been investigated for prognostic and predictive utility when considering systemic therapy, and prominent amongst these is excision repair cross-complementation group 1 (ERCC1) protein.

ERCC1 is the rate-limiting member of the nucleotide excision repair pathway (NER), one of at least 5 overlapping biochemical pathways by which altered DNA sequences can be restored to base-line. Abrogation of these pathways has been both associated with carcinogenesis [5], and targeted as a therapeutic mechanism [6]. The NER pathway functions to remove bulky DNA lesions [5], including tobacco-associated adducts formed by carcinogen exposure [7]. Mechanisms of platinum cytotoxicity include forming bulky DNA adducts leading to both inter-and intra-strand cross-link generation, which results in apoptosis unless repaired.

The critical role of ERCC1 in carcinogen and platinum adduct removal by NER has led to a number of studies reporting the relationship between ERCC1 status and survival in lung cancer patients, predominantly in NSCLC. ERCC1 has been investigated both as a prognostic biomarker, and for a predictive influence in determining benefit from platinum-directed therapy, with estimates between studies differing considerably.

We performed a systematic review and, where possible, meta-analysis of study outcomes to produce evidence-based results on the prognostic and predictive utility of ERCC1 status in lung cancer, and identify further research needs.

## Methods

Systematic review and meta-analysis was performed according to Cochrane [8], QUORUM [9], and PRISMA [10] guidelines.

### Eligibility Criteria

English language published studies were eligible if they assessed association of ERCC1 expression with survival or tumour response in NSCLC or SCLC patients. The primary outcomes of interest were overall survival (OS), event-free survival (EFS) and tumour response to chemotherapy, as defined by the contributing studies. For publications with overlapping datasets the smaller series was excluded.

### Identification of Studies

The search for studies was performed in duplicate (SP and RAH) using the electronic database PubMed (http://www.pubmed.com) until 10th August 2009. The search strategy used the keywords “lung cancer,” “NSCLC”, or “SCLC”, and “ERCC1”. Bibliographies of eligible studies, review articles and other relevant publications were also hand-searched to identify additional studies. Data from review articles, abstracts, and letters were not included.

### Data extraction

Study characteristics were extracted from the full published article and summarized in a consistent manner to aid comparison. Methodology of ERCC1 analysis was categorized, including the threshold used to dichotomize ERCC1 as “high” and “low”.

For platinum or non-platinum based chemotherapy treatment groups within each study, the log hazard ratio (log(HR)) estimate and its variance were extracted (log ratio of survival risk in ERCC1 high group versus ERCC1 low group). When not directly reported the log(HR) and its variance were estimated [11], [12], from other data, such as log rank test statistics and p values, and number of patients and events in each group, using the methods of Parmar et al [13], [14]. Where relevant effect estimates were not obtainable using the methods above, or through direct contact with corresponding authors, the study was excluded from the meta-analysis. Both unadjusted and adjusted HR estimates were sought for each study, and the choice of adjustment factors recorded.

In addition, we sought to extract the log relative risk (log(RR)) estimate and its variance indicating the log of the ratio of the risk of tumour response (response versus non-response) to platinum-based systemic therapy in the ERCC1 “high” group versus ERCC1 “low” group.

### Statistical Analysis

Direct evidence of ERCC1 as a predictive biomarker for platinum-based chemotherapy, defined by the interaction between platinum-based versus non-platinum-based chemotherapy and ERCC1 status within randomised controlled trials, was summarised where available. Indirect evidence of predictive influence was obtained by collating evidence on the prognostic effect of ERCC1 in platinum-based treated groups and non-platinum-based treated groups available separately from studies, and on the relationship between ERCC1 and radiological response to platinum-based chemotherapy.

Heterogeneity between studies was expected, hence extracted study log(HR) or log(RR) estimates and their variances were pooled using a random effects meta-analysis which accounts for such heterogeneity; estimates the average (‘summary’) HR across studies and its confidence interval (CI); and provides a prediction interval for the true hazard ratio in an individual study setting [15], [16].

The impact of between-study heterogeneity in our meta-analyses was assessed by the *I^2^* statistic [17]. *I^2^* describes the proportion of total variation in meta-analysis estimates due to between-study heterogeneity, and is measured from 0–100% with increasing I^2^ values indicating a larger impact of between-study heterogeneity in the meta-analysis.

For meta-analyses including 10 or more studies we assessed the possibility of small study effects (which indicates potential publication bias) by performing Egger's test, with a 10% significance level due to the low power of this test [18].

All statistical computations were undertaken using STATA version 10 (Stata Corporation, College Station, TX) and the modules METAN [19], and METABIAS [20]

## Results

### Eligible Studies

**Figure 1:**
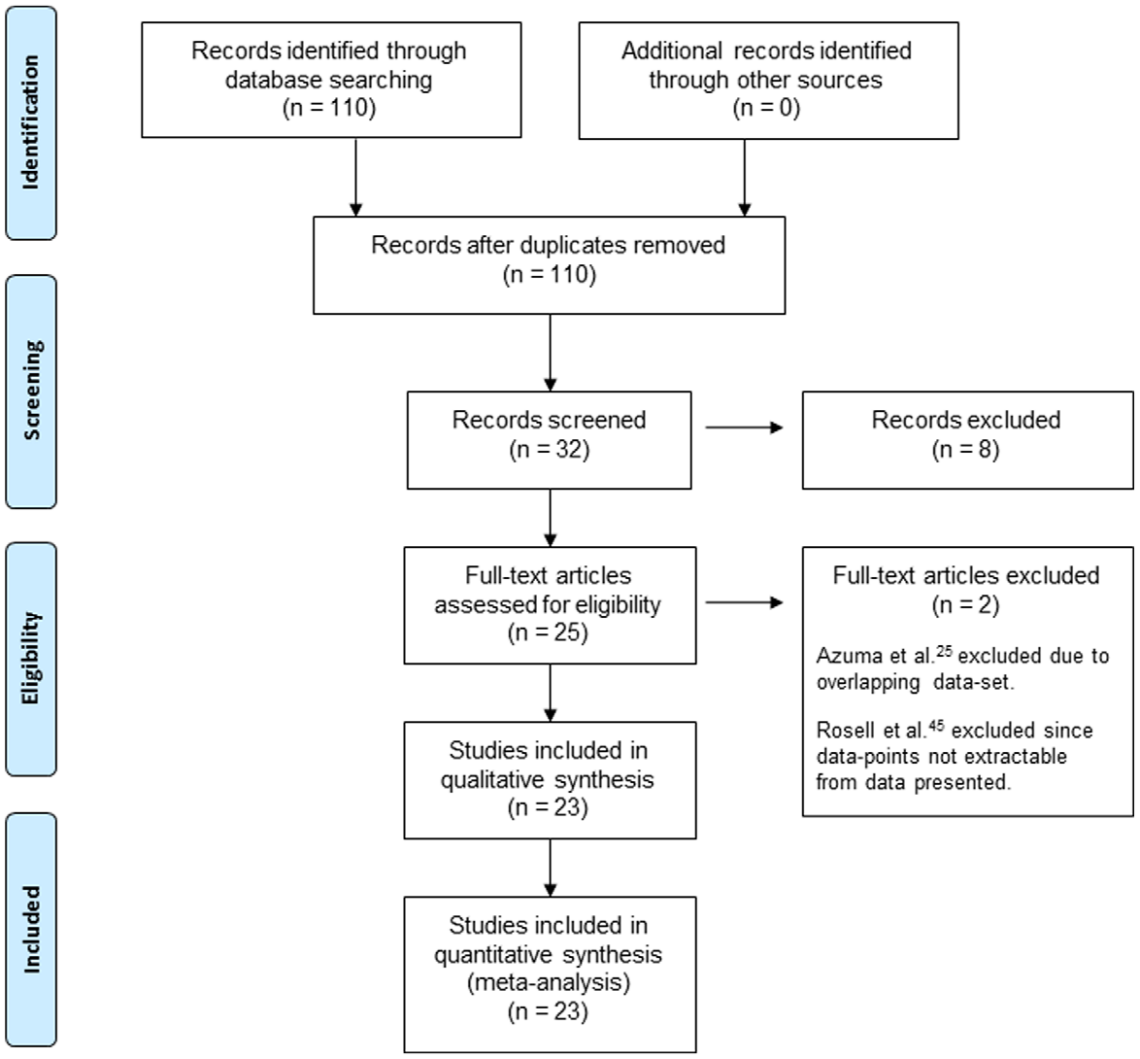
PRISMA^10^ flow chart of selection process to identify studies eligible for pooling.

We identified 25 eligible studies [21], [22], [23], [24], [25], [26], [27], [28], [29], [30], [31], [32], [33], [34], [35], [36], [37], [38], [39], [40], [41], [42], [43], [44], [45] which provided outcome data stratified by ERCC1 status (Figure 1). One of these [25] was excluded since the data set overlapped with a larger previously reported series [33], whilst in another [45] relevant effect estimates could not be obtained, leaving 23 studies from 11 countries.

In four studies [28], [32], [36], [38] outcome data were presented separately for patients who underwent different therapeutic strategies, and these groups were treated as separate datasets (Table 1). Specifically, Rosell et al. [38] stratified patients into groups who received three different chemotherapy regimes, one of which overlapped with a larger cohort [39] and was thus excluded; Olaussen et al. [36] reported separately on patients randomised to either chemotherapy or observation following resection; Okuda et al. [28] presented separate outcome data from non-randomised cohorts who underwent either peri-operative chemotherapy or surgery alone; and Fujii et al. [32] separately analysed outcomes for patients undergoing neoadjuvant chemotherapy or chemoradiotherapy, but datapoints were not extractable for the chemoradiotherapy group and this dataset was therefore excluded. Two studies [23], [27] reporting patients with SCLC presented separate outcome data by stage (limited or extensive), and these datasets were treated separately (Table 1).

**Table 1:** Summary of studies reporting ERCC1 expression and outcomes in non-small cell and small-cell lung cancer patients.

Study	No of patients*	Clinical trial	Stage	Chemotherapy	ERCC1 Method	% high ERCC1 expression
***NSCLC***						
Lord et al. [39]	56	Yes	IIIB-IV	Cisplatin/gemcitabine	RTqPCR	50
Rosell et al. [38] (A)	31	Yes	IIIB-IV	Cisplatin/gemcitabine/vinorelbine	RTqPCR	52
Rosell et al. [38] (B)	29	Yes	IIIB-IV	Gemcitabine/vinorelbine and vinorelbine/ifosfamide	RTqPCR	41
Simon et al. [37]	51	No	Resected IA-IIIB	Noneƒ	RTqPCR	∼50
Olaussen et al. [36] (A)	389	Yes	I-III	Adjuvant cisplatin/etoposide or cisplatin/vinca alkaloid	IHC	42
Olaussen et al. [36] (B)	372	Yes	I-III	None	IHC	46
Ceppi et al. [35]	61	Yes	IIIA-IV	Cisplatin/gemcitabine or gemcitabine monotherapy	RTqPCR	56
Booton et al. [31]	66	Yes	IIIA-IV	Carboplatin/docetaxel or MIC or MVP	RTqPCR	50
Zeng et al. [34]	184	No	Resected IA-IB	None	IHC	50
Rosell et al. [30]	126	No	Resected I-IIIA	None	RTqPCR	50
Azuma et al. [33]	67	No	Recurrent	Platinum doublet	IHC	43
Lee et al. [29]	130	No	Resected I-III	None	IHC	62
Fujii et al. [32] (A)	15	Yes	IIIA-IIIB	Neo-adjuvant cisplatin/irinotecan	IHC	47
Fujii et al. [32] (B)	20	Yes	IIIA-IIIB	Neo-adjuvant chemoRT; cisplatin/docetaxel	IHC	65
Okuda et al. [28] (A)	90	No	Resected I-IV	Neo-adjuvant or adjuvant platinum doublet	IHC	43
Okuda et al. [28] (B)	59	No	NS	None	IHC	34
Hwang et al. [21]	68	No	IIIA	Neo-adjuvant chemoRT; platinum doublet	IHC	46
Lee et al. [41]	50	No	IIIB/IV/recurrent	Platinum doublet	IHC	56
Azuma et al. [22]	34	No	IIB-IIIB	Concurrent chemoRT; cisplatin/docetaxel	IHC	47
Ota et al. [26]	156	No	IV	Platinum doublet	IHC	64
Wang et al. [42]	124	No	IIIB-IV	Cisplatin doublet	IHC	35
Holm et al. [43]	163	No	Inoperable IIB-IV	Carboplatin/gemcitabine	IHC	43
Jeong et al. [44]	39	No	III	ChemoRT; cisplatin doublet or triplet	IHC	31
Bartolucci et al. [24]	54	No	Resected IB-IIB	None	RTqPCR	50
***SCLC***						
Ceppi et al. [27] (A)	40	No	Extensive	Cisplatin/carboplatin and etoposide	RTqPCR	51⌋
Ceppi et al. [27] (B)	45	No	Limited	Cisplatin/carboplatin and etoposide	RTqPCR	
Lee et al. [23] (A)	37	No	Extensive	Platinum doublet	IHC	17⌋
Lee et al. [23] (B)	40	No	Limited	Platinum doublet	IHC	
Kim et al. [40]	130	No	Extensive (86%)	Platinum-based combination	IHC	28

RTqPCR, reverse transcriptase quantitative polymerase chain reaction; IHC, immunohistochemistry; MIC, mitomycin/ifosfamide/cisplatin; MVP, mitomycin/vinblastine/cisplatin; chemoRT, chemoradiotherapy; NS, not stated;

*, number of patients assessable for ERCC1 expression and overall survival;

ƒ , one patient received adjuvant chemoradiotherapy;

⌋ , % high ERCC1 expression overall (data not stated for subgroups separately).

### Study Characteristics

Characteristics of the 23 eligible studies are summarised in Table 1. All studies assessed ERCC1 expression retrospectively. Six studies [31], [32], [35], [36], [38], [39] assessed ERCC1 in tumours from unselected patients enrolled into clinical trials, whilst in the remainder patients were not accrued to a trial. The median percentage of male patients was 76%, whilst the median of the study age means was 61 years. Three studies [28], [31], [42] did not report mean age.

OS was reported in all studies, event-free survival in nine studies [21], [22], [24], [26], [30], [32], [33], [34], [38] (classified as progression-free survival [21], [22], [26], [33], disease-free survival [24], [32], [34], time to tumour progression [38], or event-free survival [30]), and response rate in 12 studies [21], [22], [23], [26], [27], [31], [32], [33], [39], [40], [41], [42]. Criteria used to assess tumour response were either Response Evaluation Criteria in Solid Tumours (RECIST) [22], [33], [40], [41], World Health Organization (WHO) [21], [23], [26], [39], [42], Eastern Cooperative Oncology Group (ECOG) [32], or unspecified [27], [31]. Sample sizes of the datasets assessed for overall survival ranged from 15 to 389 (median 54; Table 1), with data from a total of 2,726 patients available for pooling.

Of the 20 studies that reported on NSCLC, eleven [22], [26], [31], [33], [35], [38], [39], [41], [42], [43], [44] included patients with inoperable/advanced/recurrent disease (stages IIB-IV), who received either platinum-containing chemotherapy [26], [31], [33], [35], [38], [39], [41], [42], [43], platinum-containing chemoradiotherapy [25], [44], or non-platinum-containing chemotherapy [38]. Whilst in the remaining nine studies [21], [24], [28], [29], [30], [32], [34], [36], [37] patients were treated radically undergoing resection alone [24], [28], [29], [30], [34], [36], [37], or resection combined with either neoadjuvant or adjuvant cisplatin-based chemotherapy [28], [32], [36] or chemoradiotherapy [21], [32].

Chemotherapy regimens used in each study are detailed in Table 1. Fifteen studies used platinum-containing regimens, with eight using only cisplatin [25], [32], [35], [36], [38], [39], [42], [44], one only carboplatin [43], and either in six [21], [26], [28], [31], [33], [41].

### ERCC1 status assignation

ERCC1 evaluation was performed by immunohistochemistry (IHC) in 15 studies [21], [23], [25], [26], [28], [29], [32], [33], [34], [36], [40], [41], [42], [43], [44], and real-time quantitative polymerase chain reaction (RTqPCR) in the remaining eight [24], [27], [30], [31], [32], [37], [38], [39]. Samples evaluated were surgical resection specimens in eight studies [24], [28], [29], [30], [33], [34], [36], [37], biopsies of primary tumour, involved lymph nodes, or metastases in 14 studies [21], [23], [26], [27], [31], [32], [35], [38], [39], [40], [41], [42], [43], [44], or both resection and biopsy specimens [25]. ERCC1 status was assessed blinded to outcome data in the majority (17/23) of studies [21], [22], [23], [26], [27], [29], [32], [33], [35], [36], [38], [39], [40], [41], [42], [43], [44], whilst in the remaining six [24], [28], [30], [31], [34], [37] blinding information was not provided.

In the 15 studies evaluating ERCC1 expression by IHC, ten [21], [22], [23], [26], [], [32], [40], [41], [42], [43,44] evaluated biopsy specimens, three studies [28], [33], [36] used tissue from resection specimens, and the remaining two [29], [34] used a tissue microarray. All used the same monoclonal antibody (8F1), but marked heterogeneity was observed between thresholds used to dichotomise ERCC1 status. Whilst the majority (10/15) of studies assessed both staining extent and intensity, thresholds varied; one study used image analyser software to evaluate samples [34]; three studies derived a composite (H) score by multiplying extent cell-staining score (0–3; 0 = none, 1 = 1–9%, 2 = 10–49%, 3≥50%) by intensity score (0–3; 0 = none, 1 = weak, 2 = moderate, 3 = strong) with H-score above median [41], [44] or ≥2 [23] designated ERCC1 high; a further four studies used the same intensity score but a different extent score (0–1; 0 = none, 0.1 = 1–9%, 0.5 = 10–49%, 1≥50%) with H-score >1 [21], [28], [36] or >0 [43] designated ERCC1 high; one study [29] graded intensity as above, multiplied this by the percentage of cells stained and used the median H-score (10, range 0–240); whilst the remaining study [22] calculated an H-score of 0–3 (0 = no staining, 1 = faint in <10% cells, 2 = weak/moderate in >10% cells, 3 = strong in >10% cells) with ≥2 designated ERCC1 high. In the remaining five studies, percentage of cells staining was examined alone, with samples >10% [26], [40], [42], >25% [33], or above median percentage [32] designated ERCC1 high.

In the eight RTqPCR-based studies six [24], [27], [31], [35], [38], [39] used fixed tumour specimens whilst two [30], [37] used frozen material. In all but one study the deltaCt mRNA method was used, comparing gene of interest to an internal reference (APPBP2 [31], β-actin [24], [27], [35], [38], [39], or ribosomal 18S [37]). In the remaining study Rosell et al. [30] normalised to both ribosomal 18S and a commercially available calibrator sample. Again, varying thresholds were used to dichotomize ERCC1. Unbiased thresholds used included median [24], [27], [30], [31], [35], [39] (threshold (T-) value range 1.4–9.0), or approximate median [37] (T-value 50) mRNA expression, whilst in one study a maximal χ^2^ method to determine the optimal cut-off value contingent on *post hoc* outcomes was used [38].

The observed median proportion of NSCLCs with high ERCC1 expression was 46% (range 17–65%), and 50% (range 41–56%) respectively in studies using IHC and RTqPCR. In SCLC, the two studies using IHC reported 17% [23] and 28% [40] high expression, and 51% in the study using RTqPCR [27].

### Survival data extracted from studies

Of the 23 studies providing data for meta-analysis, there were 16 studies in regard OS in patients with NSCLC who received platinum-containing chemotherapy; one study in regard NSCLC patients receiving a non-platinum-containing regimen; seven studies in regard NSCLC patients undergoing surgery alone, and five studies for SCLC patients (Table 2). Although most studies (n = 23) provided unadjusted results [21], [22], [23], [24], [26], [27], [28], [29], [30], [31], [32], [33], [34], [35], [37], [38], [39], [40], [41], [44], fewer studies (n = 17) provided adjusted results [21], [22], [23], [24], [26], [27], [28], [29], [31], [33], [36], [37], [39], [41], [42], [43]. Eleven datasets were available for pooling EFS, all with unadjusted [21], [22], [24], [26], [30], [32], [33], [34], [38], and five with adjusted [21], [22], [24], [26], [33] datapoints (Table 2). Mean follow-up time data were presented by most investigators, with a median of 15 months for non-resected NSCLC (range 11–24), and 48 months for resected NSCLC (range 30–106). In the three studies investigating SCLC, mean follow-up was unreported [27], 12 months [23], and 100 months [40]

**Table 2:** Results of survival analyses by individual study.

	Overall Survival				Event Free Survival			
Study	Univariate		Multivariate		Univariate		Multivariate	
	HR	95% CI	HR	95% CI	HR	95% CI	HR	95% CI
***NSCLC No Chemotherapy***								
Simon et al. [37]	0.34	0.14–0.83	0.24	0.08–0.77	—	—	—	—
Olaussen et al. [36] (B)	—	—	0.66	0.49–0.90	—	—	—	—
Zeng et al. [34]	0.54*	0.34–0.86*	—	—	0.64*	0.38–1.10*	—	—
Rosell et al. [30]	0.96*	0.53–1.74*	—	—	0.96*	0.51–1.79*	—	—
Lee et al. [29]	0.61*	0.38–0.99*	0.60	0.36–1.00	—	—	—	—
Okuda et al. [28] (B)	1.68*	0.69–4.06*	1.62	0.71–3.70	—	—	—	—
Bartolucci et al. [24]	2.07*	0.94–4.54*	1.17	0.62–2.21	1.71*	0.78–3.76*	1.15	0.56–2.37
***NSCLC Platinum Treated***								
Lord et al. [39]	2.39*	1.24–4.59*	3.13	1.41–7.14	—	—	—	—
Rosell et al. [38] (A)	0.59*	0.26–1.30*	—	—	0.92*	0.45–1.91*	—	—
Olaussen et al. [36] (A)	—	—	1.16	0.86–1.56	—	—	—	—
Ceppi et al. [35]	2.28*	1.32–3.94*	—	—	—	—	—	—
Booton et al. [31]	0.91*	0.45–1.85*	0.96	0.92–1.00	—	—	—	—
Azuma et al. [33]	2.99*	1.60–5.59*	1.65	1.21–2.28	2.22*	1.24–3.97*	1.37	1.07–1.76
Fujii et al. [32] (A)	1.48*	0.45–4.82*	—	—	1.65*	0.48–5.71*	—	—
Fujii et al. [32] (B)	1.85*	0.30–11.65*	—	—	0.62*	0.15–2.65*	—	—
Okuda et al. [28] (A)	2.43*	1.28–4.61*	2.31	1.24–4.31	—	—	—	—
Hwang et al. [21]	2.14*	1.17–3.93*	2.07	1.03–4.17	1.77*	0.97–3.23*	1.57	0.83–2.98
Lee et al. [41]	1.79*	0.99–3.25*	3.16	1.54–6.46	—	—	—	—
Azuma et al. [22]	1.73*	0.74–4.09*	2.41	0.86–6.76	2.79*	1.29–6.03*	3.97	1.41–11.23
Ota et al. [26]	1.46*	1.04–2.05*	1.33	0.93–1.92	0.69*	0.47–1.02*	1.22	0.79–1.85
Wang et al. [42]	—	—	1.72	1.16–2.53	—	—	—	—
Holm et al. [43]	—	—	1.24	1.01–1.51	—	—	—	—
Jeong et al. [44]	0.64*	0.32–1.28*	—	—	—	—	—	—
***NSCLC Non-Platinum Treated***								
Rosell et al. [38] (B)	0.77*	0.34–1.74*	—	—	1.08*	0.52–2.26*	—	—
***SCLC***								
Ceppi et al. [27] (All patients)	1.46*	0.94–2.26*	—	—	—	—	—	—
Ceppi et al. [27] (B; LS)	2.19*	1.19–4.04*	2.06	1.18–4.38	—	—	—	—
Lee et al. [23] (A; ES)	0.82*	0.37–1.79*	1.07	0.46–2.49	—	—	—	—
Lee et al. [23] (B; LS)	3.66*	1.26–10.60*	2.80	1.02–2.39	—	—	—	—
Kim et al. [40]	0.90*	0.61–1.35*	—	—	—	—	—	—

HRs and associated 95% CIs are given as quoted unless stated otherwise, (—) indicates not assessed;

*estimated result from data presented in paper using methods of Palmer et al. (REF);

HR, hazard ratio; CI, confidence interval; LS, limited stage; ES, extensive stage.

### Direct evidence for predictive influence of ERCC1

Only one study [36], based on a subset of 761 of 1,867 trial patients, provided direct evidence on ERCC1 as a predictive biomarker in the form of an interaction between randomised treatment and ERCC1 status which was statistically significant (p = 0.009), with adjuvant cisplatin-based chemotherapy prolonging survival compared with observation in patients with ERCC1-negative tumours (adjusted HR = 0.65; 95%CI: 0.50–0.86) but not in patients with ERCC1-positive tumours (adjusted HR = 1.14; 95%CI: 0.84–1.55).

### Indirect evidence for predictive influence of ERCC1

#### i) Relationship between ERCC1 status and survival in NSCLC without systemic therapy

**Figure 2:**
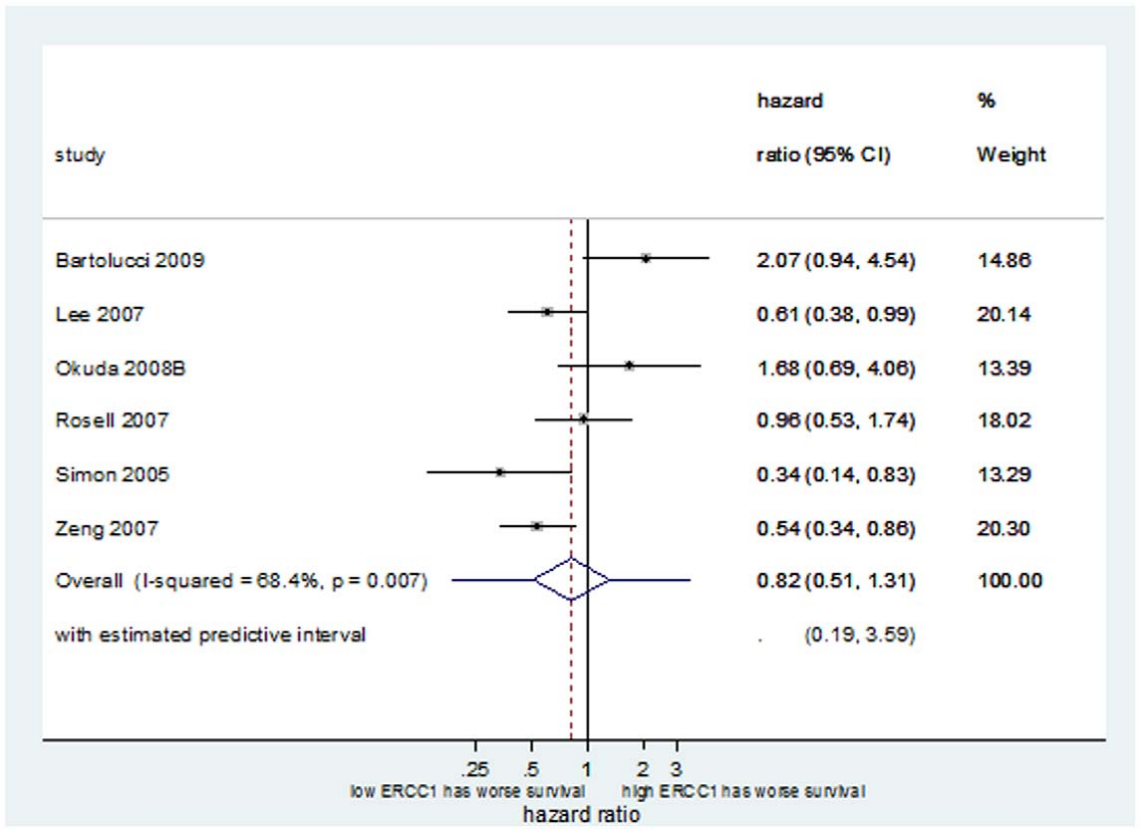
Forest plot showing the meta-analysis of unadjusted hazard ratio estimates for OS in NSCLC patients not treated with chemotherapy.

Seven datasets [24], [28], [29], [30], [34], [36], [37] assessing 896 patients, with high ERCC1 expression observed in 48% of tumours, were available for pooling estimates of survival in patients who underwent surgery alone without systemic therapy. Neither the meta-analysis of unadjusted nor meta-analysis of adjusted estimates provided evidence that ERCC1 status has prognostic value for either OS or EFS in these patients (Table 3 and Figure 2).

**Table 3:** Meta-analysis results.

	Meta-analysis of unadjusted estimates					Meta-analysis of adjusted estimates				
Studies	n studies	PooledHR	Pooled95% CIs	I^2^(%)	95% prediction interval	nstudies	PooledHR	Pooled95% CIs	I^2^(%)	95% prediction interval
NSCLC platinum treated, OS	13	1.61	1.23–2.10	52	0.71–3.62	11	1.57	1.24–1.99	83	0.73–3.37
NSCLC platinum treated, EFS	7	1.36	0.83–2.21	82	0.30–6.05	4	1.46	1.09–1.95	32	0.56–3.81
NSCLC no chemotherapy, OS	6	0.82	0.51–1.31	68	0.19–3.59	5	0.75	0.49–1.16	61	0.19–2.92
NSCLC no chemotherapy, EFS	3	0.96	0.56–1.63	51	0.01–226	—	—	—	—	—
SCLC all patients, OS	3	1.33	0.85–2.09	62	0.01–190	—	—	—	—	—
SCLC limited stage, OS	2	2.49	1.86–4.23	0	—	2	2.26	1.30–3.91	0	—

OS, overall survival; EFS, event-free survival; HR, hazard ratio; CI, confidence interval.

#### ii) Relationship between ERCC1 status and survival in NSCLC with platinum-based systemic therapy

**Figure 3:**
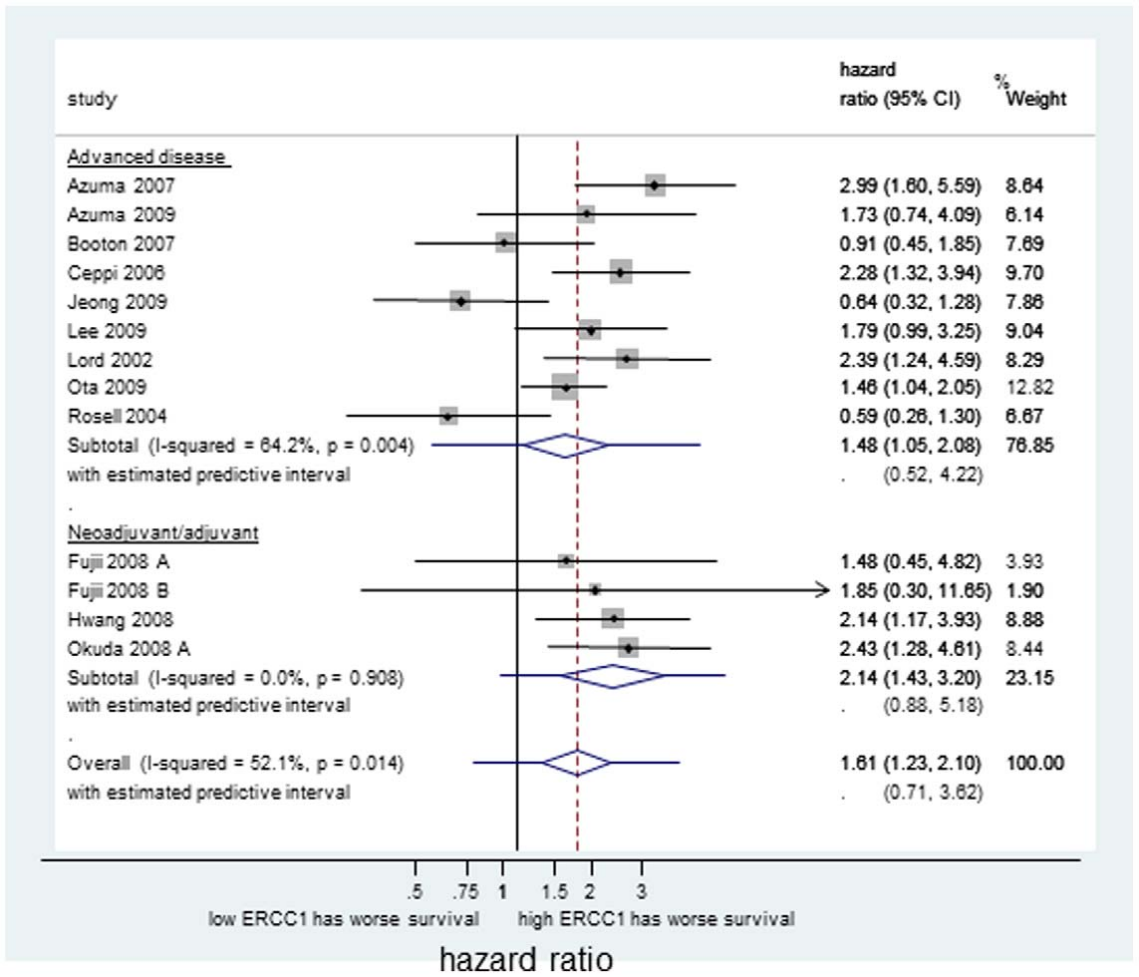
Forest plot showing the meta-analysis of unadjusted hazard ratio estimates for OS in NSCLC patients receiving platinum-based chemotherapy.

OS HR estimates obtained from 1,391 NSCLC patients who received platinum were available for meta-analysis, 640 (46%) of whom had tumours with high ERCC1 expression. Meta-analysis of the unadjusted estimates indicated a significantly poorer OS in patients with high ERCC1 expression (average HR = 1.61, 95%CI: 1.23–2.10). This effect was maintained when pooling adjusted estimates (average HR = 1.57, 95%CI: 1.24–1.99), and was observed in patients treated in both the adjuvant and advanced disease settings (Figure 3). However, large heterogeneity was observed in both unadjusted and adjusted analyses (I^2^ = 52% and I^2^ = 83%, respectively), resulting in wide prediction intervals for the prognostic effect in an individual clinical setting, and whilst there was no evidence of small study effects using unadjusted estimates (Egger's test: p = 0.72), there was clear evidence of such using adjusted estimates (Egger's test: p<0.0001). Meta-analysis for EFS indicated a relationship with ERCC1 status using adjusted estimates (average HR = 1.46, 95%CI:1.09–1.95, I^2^ = 32%), but not with unadjusted (Table 3), and investigation of small study effects was not possible due to the small number of studies.

#### iii) Relationship between ERCC1 status and survival in SCLC

Five studies were available in regard SCLC patients receiving platinum-based chemotherapy (Table 2), contributing data on 292 patients, of whom 92 (32%) had high ERCC1 expressing tumours. Meta-analysis of neither unadjusted nor adjusted estimates indicated an association between OS and high ERCC1 expression (Table 3). In an exploratory analysis stratifying by stage of disease, there was evidence of association between high ERCC1 expression and poor OS in limited stage patients, although this was based on only 2 studies.

#### iv) Relationship between ERCC1 status and tumour response

**Figure 4:**
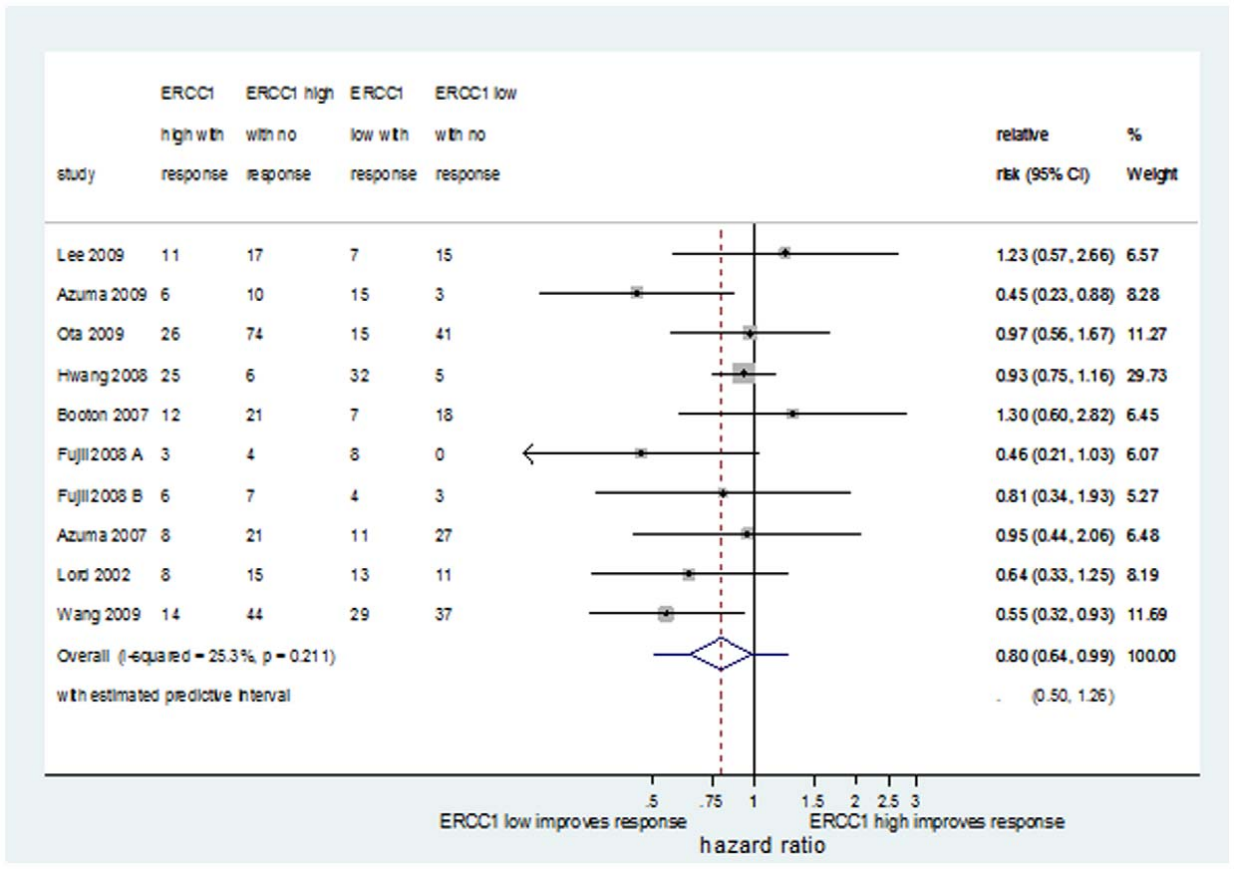
Forest plot showing the meta-analysis of unadjusted relative risk estimates for response rate in NSCLC patients receiving platinum-based chemotherapy.

Tumour response stratified by ERCC1 expression was reported by ten [21], [22], [26], [31], [32], [33], [39], [41], [42] NSCLC data-sets comprising 656 patients, 328 (50%) of whom had high expression. All patients were treated with platinum containing chemotherapy. There was evidence that high ERCC1 was associated with a reduced response to platinum (average RR = 0.80; 95%CI:0.64–0.99, Figure 4) with moderate heterogeneity (I^2^ = 25.3%) and no evidence of small study effects (Egger's test: p = 0.36). Meta-analysis of three studies in SCLC (292 patients), also provided evidence of a trend towards increased response to platinum-containing chemotherapy in high ERCC1 expressing tumours (average RR = 1.14; 95% CI: 0.99–1.31, p = 0.08; I^2^ = 0%), although this was not statistically significant.

## Discussion

Somatic molecular phenotype has become well established as a key determinant in NSCLC in terms of both outcome and efficacy of systemic therapy [46], [47], [48]. Previous studies have suggested that somatic ERCC1 expression level is both prognostic in lung cancer, and predictive of outcome to platinum-based chemotherapy. Indeed, trials have already reported randomising patients contingent on somatic ERCC1 status [49], [50], with others actively accruing. However, our analysis has shown that many of the published series assessing the ERCC1 utility have been small in size, reported conflicting outcomes, and used widely differing expression methodology and, in particular, threshold levels.

Only one study [36] directly assessed the predictive utility of ERCC1 status for platinum-based chemotherapy in NSCLC, and this was retrospective in design and in a subgroup of patients recruited to a clinical trial. A relationship between ERCC1 and survival in platinum-treated patients but not in non-platinum treated patients provides indirect evidence that ERCC1 is predictive, although the robustness of this conclusion is compromised due to the non-randomised nature of the treatment groups. Based on data from NSCLC patients untreated with systemic therapy, our analyses do not support the hypothesis that ERCC1 expression is prognostic in NSCLC. However, tentative evidence that, on average, high ERCC1 expression identifies poorer survival in NSCLC patients treated with platinum-based chemotherapy, provided indirect evidence of a predictive influence, although this may be merely a prognostic effect in this patient group. The lower likelihood of response to platinum-based chemotherapy in high ERCC1 expressing tumours observed further supports the notion that ERCC1 might be predictive of lack of platinum benefit.

Our study has identified several methodological weaknesses that must be addressed. Firstly, we observed notable variation in cut-offs in ERCC1 expression, which account in part for heterogeneity observed. Second, there has been considerable debate as to whether the 8F1 antibody clone specifically binds ERCC1 alone or other non-ERCC1 epitopes [51], [52]. Third, it is unclear whether ERCC1 expression analysis by IHC and RTqPCR stratify patients into similar groups [34], and in the absence of a proven correlation between these two methods we recommend the use of IHC since it is more readily available and more easily standardised across laboratories. For ERCC1 to be a useful predictive biomarker in clinical practice, a single clearly defined protocol needs to be developed and validated to allow comparison of outcomes across studies. Inadequate sample size was also a frequent problem in the studies included in our analyses, with only eight of the 23 studies reporting outcomes from over 100 patients. Whilst pooling data may in part address deficiencies in individual study sample sizes, smaller studies are more likely to generate heterogeneity, as we observed. Several independent studies, each an order of magnitude greater than most published series, are required to allow accurate estimation of the true associations between ERCC1 expression and outcomes in lung cancer patients, particularly in SCLC.

Strengths of our study include the analysis of both survival and chemotherapy response endpoints, the separate meta-analysis of studies in which patients did or did not receive chemotherapy allowing an assessment of both predictive and indirect prognostic influences, calculation of prediction intervals to estimate the range of potential effects in any one individual study, and extensive comparison of study methodologies. In contrast, a similar recently published meta-analysis [53] made no assessment of the influence of ERCC1 status in patients who did not receive chemotherapy or SCLC patients, did not account for adjusting and made no assessment of methodological differences between contributing studies, failed to use the published HR and CIs for one study which compromised assessment of publication bias, used fixed effect meta-analyses despite significant between-study heterogeneity, and included potentially overlapping datasets. In our study we did not assess the quality of the primary studies, however quality assessment tools for examining prognostic and predictive biomarker studies do not currently exist, and are only beginning to be discussed for prognosis studies in general [54].

Large heterogeneity, methodological concerns, and potential for publication bias revealed by our analyses indicate that although ERCC1 shows considerable promise as a predictive biomarker in platinum-treated NSCLC patients, it is not ready for ‘prime-time’. International consensus is urgently required to mandate homogeneous ERCC1 assessment methodology, as are prospective trials sufficiently powered to detect an interaction between platinum chemotherapy and ERCC1 expression, and in the interim, initiation of large, prospectively planned individual patient data meta-analyses. Until then, ERCC1 expression should not be routinely used in clinical decision-making.

## References

[1] Jemal A, Siegel R, Ward E, Hao Y, Xu J (2009). Cancer statistics, 2009.. CA Cancer J Clin.

[2] Smit EF, van Meerbeeck JP, Lianes P, Debruyne C, Legrand C (2003). Three-arm randomized study of two cisplatin-based regimens and paclitaxel plus gemcitabine in advanced non-small-cell lung cancer: a phase III trial of the European Organization for Research and Treatment of Cancer Lung Cancer Group–EORTC 08975.. J Clin Oncol.

[3] Yau T, Ashley S, Popat S, Norton A, Matakidou A (2006). Time and chemotherapy treatment trends in the treatment of elderly patients (age >/ = 70 years) with small cell lung cancer.. Br J Cancer.

[4] Ardizzoni A, Boni L, Tiseo M, Fossella FV, Schiller JH (2007). Cisplatin- versus carboplatin-based chemotherapy in first-line treatment of advanced non-small-cell lung cancer: an individual patient data meta-analysis.. J Natl Cancer Inst.

[5] Friedberg EC (2003). DNA damage and repair.. Nature.

[6] Helleday T, Petermann E, Lundin C, Hodgson B, Sharma RA (2008). DNA repair pathways as targets for cancer therapy.. Nat Rev Cancer.

[7] Phillips DH (2002). Smoking-related DNA and protein adducts in human tissues.. Carcinogenesis.

[8] Alderson P, Green S, Higgins JPT (2004). Cochrane Reviewer's Handbook 4.2.2. The Cochrane Library.

[9] Moher D, Cook DJ, Eastwood S, Olkin I, Rennie D (1999). Improving the quality of reports of meta-analyses of randomised controlled trials: the QUOROM statement. Quality of Reporting of Meta-analyses.. Lancet.

[10] Moher D, Liberati A, Tetzlaff J, Altman DG (2009). Preferred reporting items for systematic reviews and meta-analyses: the PRISMA statement.. PLoS Med.

[11] Altman DG, De Stavola BL, Love SB, Stepniewska KA (1995). Review of survival analyses published in cancer journals.. Br J Cancer.

[12] Riley RD, Abrams KR, Sutton AJ, Lambert PC, Jones DR (2003). Reporting of prognostic markers: current problems and development of guidelines for evidence-based practice in the future.. Br J Cancer.

[13] Parmar MK, Torri V, Stewart L (1998). Extracting summary statistics to perform meta-analyses of the published literature for survival endpoints.. Stat Med.

[14] Williamson PR, Smith CT, Hutton JL, Marson AG (2002). Aggregate data meta-analysis with time-to-event outcomes.. Stat Med.

[15] Riley RD, Higgins JP, Deeks JJ (2011). Interpretation of random effects meta-analyses.. BMJ.

[16] Higgins JP, Thompson SG, Spiegelhalter DJ (2009). A re-evaluation of random-effects meta-analysis.. J R Stat Soc Ser A Stat Soc.

[17] Higgins JP, Thompson SG (2002). Quantifying heterogeneity in a meta-analysis.. Stat Med.

[18] Sterne JAC, Egger M, Moher D, Higgins JPT, Green S (2008). Addressing reporting biases.. Cochrane Handbook for Systematic Reviews of Interventions.

[19] Bradburn MJ, Deeks JJ, Altman DG (Stata Corporation, 1999). Metan-an alternative meta-analysis command.. Stata Technical Bulletin.

[20] Steichen TJ (1998). SBE19: Tests for publication bias in meta-analysis.. Stata Technical Bulletin.

[21] Hwang IG, Ahn MJ, Park BB, Ahn YC, Han J (2008). ERCC1 expression as a prognostic marker in N2(+) nonsmall-cell lung cancer patients treated with platinum-based neoadjuvant concurrent chemoradiotherapy.. Cancer.

[22] Azuma K, Sasada T, Kawahara A, Hattori S, Kinoshita T (2009). Expression of ERCC1 and class III beta-tubulin in non-small cell lung cancer patients treated with a combination of cisplatin/docetaxel and concurrent thoracic irradiation.. Cancer Chemother Pharmacol.

[23] Lee HW, Han JH, Kim JH, Lee MH, Jeong SH (2008). Expression of excision repair cross-complementation group 1 protein predicts poor outcome in patients with small cell lung cancer.. Lung Cancer.

[24] Bartolucci R, Wei J, Sanchez JJ, Perez-Roca L, Chaib I (2009). XPG mRNA expression levels modulate prognosis in resected non-small-cell lung cancer in conjunction with BRCA1 and ERCC1 expression.. Clin Lung Cancer.

[25] Azuma K, Sasada T, Kawahara A, Takamori S, Hattori S (2009). Expression of ERCC1 and class III beta-tubulin in non-small cell lung cancer patients treated with carboplatin and paclitaxel.. Lung Cancer.

[26] Ota S, Ishii G, Goto K, Kubota K, Kim YH (2009). Immunohistochemical expression of BCRP and ERCC1 in biopsy specimen predicts survival in advanced non-small-cell lung cancer treated with cisplatin-based chemotherapy.. Lung Cancer.

[27] Ceppi P, Longo M, Volante M, Novello S, Cappia S (2008). Excision repair cross complementing-1 and topoisomerase IIalpha gene expression in small-cell lung cancer patients treated with platinum and etoposide: a retrospective study.. J Thorac Oncol.

[28] Okuda K, Sasaki H, Dumontet C, Kawano O, Yukiue H (2008). Expression of excision repair cross-complementation group 1 and class III beta-tubulin predict survival after chemotherapy for completely resected non-small cell lung cancer.. Lung Cancer.

[29] Lee KH, Min HS, Han SW, Oh DY, Lee SH (2008). ERCC1 expression by immunohistochemistry and EGFR mutations in resected non-small cell lung cancer.. Lung Cancer.

[30] Rosell R, Skrzypski M, Jassem E, Taron M, Bartolucci R (2007). BRCA1: a novel prognostic factor in resected non-small-cell lung cancer.. PLoS One.

[31] Booton R, Ward T, Ashcroft L, Morris J, Heighway J (2007). ERCC1 mRNA expression is not associated with response and survival after platinum-based chemotherapy regimens in advanced non-small cell lung cancer.. J Thorac Oncol.

[32] Fujii T, Toyooka S, Ichimura K, Fujiwara Y, Hotta K (2008). ERCC1 protein expression predicts the response of cisplatin-based neoadjuvant chemotherapy in non-small-cell lung cancer.. Lung Cancer.

[33] Azuma K, Komohara Y, Sasada T, Terazaki Y, Ikeda J (2007). Excision repair cross-complementation group 1 predicts progression-free and overall survival in non-small cell lung cancer patients treated with platinum-based chemotherapy.. Cancer Sci.

[34] Zheng Z, Chen T, Li X, Haura E, Sharma A (2007). DNA synthesis and repair genes RRM1 and ERCC1 in lung cancer.. N Engl J Med.

[35] Ceppi P, Volante M, Novello S, Rapa I, Danenberg KD (2006). ERCC1 and RRM1 gene expressions but not EGFR are predictive of shorter survival in advanced non-small-cell lung cancer treated with cisplatin and gemcitabine.. Ann Oncol.

[36] Olaussen KA, Dunant A, Fouret P, Brambilla E, Andre F (2006). DNA repair by ERCC1 in non-small-cell lung cancer and cisplatin-based adjuvant chemotherapy.. N Engl J Med.

[37] Simon GR, Sharma S, Cantor A, Smith P, Bepler G (2005). ERCC1 expression is a predictor of survival in resected patients with non-small cell lung cancer.. Chest.

[38] Rosell R, Danenberg KD, Alberola V, Bepler G, Sanchez JJ (2004). Ribonucleotide reductase messenger RNA expression and survival in gemcitabine/cisplatin-treated advanced non-small cell lung cancer patients.. Clin Cancer Res.

[39] Lord RV, Brabender J, Gandara D, Alberola V, Camps C (2002). Low ERCC1 expression correlates with prolonged survival after cisplatin plus gemcitabine chemotherapy in non-small cell lung cancer.. Clin Cancer Res.

[40] Kim YH, Ishii G, Goto K, Ota S, Kubota K (2009). Expression of breast cancer resistance protein is associated with a poor clinical outcome in patients with small-cell lung cancer.. Lung Cancer.

[41] Lee HW, Choi YW, Han JH, Kim JH, Jung JH (2009). Expression of excision repair cross-complementation group 1 protein predicts poor outcome in advanced non-small cell lung cancer patients treated with platinum-based doublet chemotherapy.. Lung Cancer.

[42] Wang X, Zhao J, Yang L, Mao L, An T (2009). Positive expression of ERCC1 predicts a poorer platinum-based treatment outcome in Chinese patients with advanced non-small-cell lung cancer.. Med Oncol.

[43] Holm B, Mellemgaard A, Skov T, Skov BG (2009). Different impact of excision repair cross-complementation group 1 on survival in male and female patients with inoperable non-small-cell lung cancer treated with carboplatin and gemcitabine.. J Clin Oncol.

[44] Jeong SH, Jung JH, Han JH, Kim JH, Choi YW (2009). Expression of Bcl-2 predicts outcome in locally advanced non-small cell lung cancer patients treated with cisplatin-based concurrent chemotherapy.. Lung Cancer.

[45] Rosell R, Felip E, Taron M, Majo J, Mendez P (2004). Gene expression as a predictive marker of outcome in stage IIB-IIIA-IIIB non-small cell lung cancer after induction gemcitabine-based chemotherapy followed by resectional surgery.. Clin Cancer Res.

[46] Tsao MS, Sakurada A, Cutz JC, Zhu CQ, Kamel-Reid S (2005). Erlotinib in lung cancer - molecular and clinical predictors of outcome.. N Engl J Med.

[47] Mok TS, Wu YL, Thongprasert S, Yang CH, Chu DT (2009). Gefitinib or carboplatin-paclitaxel in pulmonary adenocarcinoma.. N Engl J Med.

[48] Bang YJ, Kwak EL, Shaw AT, Camidge DR, Iafrate AS (2010). Clinical activity of the oral ALK inhibitor PF-02341066 in ALK-positive patients with non-small cell lung cancer (NSCLC).. Journal of Clinical Oncology.

[49] Cobo M, Isla D, Massuti B, Montes A, Sanchez JM (2007). Customizing cisplatin based on quantitative excision repair cross-complementing 1 mRNA expression: a phase III trial in non-small-cell lung cancer.. J Clin Oncol.

[50] Simon G, Sharma A, Li X, Hazelton T, Walsh F (2007). Feasibility and efficacy of molecular analysis-directed individualized therapy in advanced non-small-cell lung cancer.. J Clin Oncol.

[51] Niedernhofer LJ, Bhagwat N, Wood RD (2007). ERCC1 and non-small-cell lung cancer.. N Engl J Med.

[52] Bhagwat NR, Roginskaya VY, Acquafondata MB, Dhir R, Wood RD (2009). Immunodetection of DNA repair endonuclease ERCC1-XPF in human tissue.. Cancer Res.

[53] Chen S, Zhang J, Wang R, Luo X, Chen H (2010). The platinum-based treatments for advanced non-small cell lung cancer, is low/negative ERCC1 expression better than high/positive ERCC1 expression? A meta-analysis.. Lung Cancer.

[54] Hayden JA, Cote P, Bombardier C (2006). Evaluation of the quality of prognosis studies in systematic reviews.. Ann Intern Med.

